# The Complex Fibrinogen Interactions of the *Staphylococcus aureus* Coagulases

**DOI:** 10.3389/fcimb.2019.00106

**Published:** 2019-04-16

**Authors:** Sheila Thomas, Wen Liu, Srishtee Arora, Vannakambodi Ganesh, Ya-Ping Ko, Magnus Höök

**Affiliations:** Center for Infectious and Inflammatory Diseases, Institute of Biosciences and Technology, Texas A&M University Health Science Center, Houston, TX, United States

**Keywords:** vWbp, Coa, fibrinogen, protein-protein interaction, apparent affinity, Fg β-chain, *Staphylococcus aureus*

## Abstract

The two coagulases, von Willebrand factor binding protein (vWbp) and Coagulase (Coa), are critical virulence factors in several animal models of invasive *Staphylococcus aureus* (*S. aureus*) infections. These proteins are part of an intricate system of proteins that *S. aureus* uses to assemble a fibrinogen (Fg)/fibrin protective shield surrounding itself. This shield allows the microorganism to evade clearance by the host phagocytic cells. The coagulases can non-proteolytically activate the zymogen prothrombin to convert Fg to fibrin and promote the Fg/fibrin shield formation. The coagulases also bind directly to Fg and the interaction between Coa and Fg has been previously characterized in some detail. However, the mechanism(s) by which vWbp interacts with Fg remains unclear. Here, we show that vWbp and Coa have distinct interactions with Fg, despite being structurally similar. Coa binds with a significantly higher affinity to soluble Fg than to Fg coated on a plastic surface, whereas vWbp demonstrates no preference between the two forms of Fg. The two coagulases appear to target different sites on Fg, as they do not compete with each other in binding to Fg. Similar to Coa, both the N- and C-terminal halves of vWbp (vWbp-N, vWbp-C, respectively) harbor Fg-binding activities. The higher affinity Fg-binding activity resides in vWbp-N; whereas, the C-terminal region of Coa encompasses the major Fg-binding activity. Peptides constituting the previously identified Coa/Efb^1^ Fg-binding motif fail to inhibit vWbp-C from binding to Fg, indicating that vWbp-C lacks a functional homolog to this motif. Interestingly, the N-terminal prothrombin-binding domains of both coagulases recognize the Fg β-chain, but they appear to interact with different sequence motifs in the host protein. Collectively, our data provide insight into the complex interactions between Fg and the *S. aureus* coagulases.

## Introduction

The lack of effective vaccines and treatment options make infections caused by antibiotic resistant *Staphylococcus aureus* (*S. aureus*) a worldwide health concern (Fowler and Proctor, 2014). In 2017, the World Health Organization listed *S. aureus*, methicillin-resistant, vancomycin-intermediate and resistant strains as organisms for which novel strategies are desperately needed (Tacconelli and Magrini, 2017). Targeting critical staphylococcal virulence factors in the design of new therapeutics represents a strategy currently being explored by investigators in the field. In this approach, a detailed molecular understanding of the mechanisms of action of relevant virulence factors is required.

*S. aureus* has evolved to hijack host fibrinogen (Fg) and form a Fg/fibrin shield surrounding the organism, which protects bacteria from clearance. *S. aureus* does so by expressing at least a dozen Fg-binding proteins that can be divided into two groups: a group of cell wall-anchored proteins of the MSCRAMM (Microbial Surface Components Recognizing Adhesive Matrix Molecules) type and a group of secreted proteins that are referred to as the SERAMs (Secretable Expanded Repertoire Adhesive Molecules) (Foster and Höök, 1998; Harraghy et al., 2008). These two groups of Fg-binding staphylococcal proteins likely work in concert to assemble the Fg/fibrin shield (Guggenberger et al., 2012; Ko et al., 2013; Thomer et al., 2016a).

Fibrinogen (Fg), a 340 kDa host plasma glycoprotein, is comprised of three pairs of non-identical chains; the Aα, Bβ and γ-chains (Mosesson, 2005). The protein is best known for its role in the blood coagulation cascade where Fg is cleaved by the serine protease, thrombin, to generate fibrin. Fibrin spontaneously assembles the core of the blood clot to repress further bleeding (Weisel and Litvinov, 2017). In addition, Fg can modulate innate immunity via interactions with the leukocyte integrin, α_M_β_2_ (Rubel et al., 2001).

The two *S. aureus* SERAMs Coagulase (Coa) and von Willebrand factor-binding protein (vWbp) can induce blood coagulation by activating prothrombin. These coagulases are critical virulence factors that contribute to the pathogenicity of the microorganism in several animal models of *S. aureus* infections (Tager, 1974; Bjerketorp et al., 2002, 2004; Panizzi et al., 2004). For example, vWbp or Coa-deficient *S. aureus* mutants showed attenuated virulence compared to the wild-type strain in septicemia and kidney abscess models (Cheng et al., 2010).

The signature feature of *S. aureus* coagulases is their ability to non-proteolytically activate the zymogen, prothrombin (Thomer et al., 2016b). The protein structure and its interactions with prothrombin and Fg are characterized in some detail for Coa. The 2.2-Å crystal structure of the N-terminal half of Coa in complex with prothrombin revealed that the N-terminal half of Coa is composed of a D_1_D_2_ domain in which each D unit forms a three-helix bundle (Friedrich et al., 2003; Panizzi et al., 2006a). The D_1_D_2_ domain binds directly to the inactive zymogen (Friedrich et al., 2003). Activation of prothrombin requires the insertion of the two N-terminal amino acid residues of Coa; Ile^1^-Val^2^, into the Ile^16^ pocket of prothrombin (Kawabata et al., 1985; Friedrich et al., 2003; Panizzi et al., 2006b). Subsequently, the complex undergoes a conformational change, resulting in an active Coa-prothrombin complex, termed staphylothrombin that is capable of cleaving Fg (Panizzi et al., 2006b).

High resolution crystal structures of vWbp as an apo-protein or in complex with prothrombin are not available. However, structure prediction programs suggest that the N-terminal half of vWbp, which shares a 30% amino acid identity to that of the N-terminal region of Coa (Bjerketorp et al., 2004), forms a structure similar to that of the D_1_D_2_ domain of Coa (Kroh et al., 2009). Activation of prothrombin by vWbp is similar to the Coa mechanism in that the N-terminal residues, Val^1^-Val^2^ of vWbp, are required (Kroh et al., 2009). Thus, vWbp and Coa demonstrate multiple similarities in their coagulation induction function.

The functional and structural similarities of the two *S. aureus* coagulases may give the impression that the two proteins are indistinguishable. However, a closer examination reveals significant differences. Although the C-terminal halves of Coa and vWbp are both predicted to be unordered (Friedrich et al., 2003; Kroh and Bock, 2012), there are substantial differences at the amino acid sequence level (Kroh and Bock, 2012; McAdow et al., 2012b; Ko and Flick, 2016; Liesenborghs et al., 2018). The C-terminal part of Coa is comprised of a linker region and a tandemly repeated Fg-binding motif (Ko and Flick, 2016). This Fg-binding motif is not present in vWbp (McAdow et al., 2012b; Ko and Flick, 2016). Instead, the C-terminal region of vWbp harbors a unique von Willebrand factor-binding motif of 26 amino acid residues (Bjerketorp et al., 2002) that enables vWbp to bind to von Willebrand factor (vWF) and mediate *S. aureus* adherence to the vessel wall of activated endothelial cells (Claes et al., 2014). These differences demonstrate that the C-terminal halves of Coa and vWbp have distinct functions (Ko and Flick, 2016).

The coagulases also differ from each other in their affinities to prothrombin and Fg. Intact Coa appears to have a higher affinity for both prothrombin (5 nM) and Fg (33 nM) than that observed for vWbp (98 nM; 271 nM, respectively) (Cheng et al., 2010; McAdow et al., 2012b; Liesenborghs et al., 2018). Activation of prothrombin by Coa is independent of Fg, whereas, vWbp activates prothrombin by a “hysteretic conformational” mechanism (Kroh et al., 2009), where Fg is required for full activation. These observations indicate that vWbp and Coa interact with Fg by different mechanisms.

The Fg interaction with Coa has been characterized in some detail (Ko and Flick, 2016; Ko et al., 2016). The major Fg-binding motif is located to the tandem repeat domain, in which a high affinity 27 amino acid long binding motif was identified (Ko et al., 2016). Attempts to locate the Fg-binding activity of vWbp gave apparently contradicting results (McAdow et al., 2012a; Thomer et al., 2013). Using an ELISA-type binding assay, McAdow et al. showed that immobilized C-terminal vWbp bound to soluble Fg, suggesting that the C-terminal half of vWbp harbors Fg-binding activity (McAdow et al., 2012a). However, affinity chromatography, in which vWbp was coupled to a Strep-Tactin column and human plasma was passed over the column, revealed that the Fg-binding region was within the D_1_D_2_ domain and not at the C-terminal half (Thomer et al., 2013). Thereby, the Fg interaction with vWbp remains unclear.

The goal of this study is to clarify the interactions between fibrinogen and the two coagulases, particularly by characterizing the Fg-binding activity of vWbp. Our study reveals that both vWbp and Coa contain Fg binding activities in both their N- and C-terminal sections, but target different sites in Fg. The differences observed between the two coagulases suggest that vWbp binds Fg by a novel mechanism that may be important for vWbp's biological function.

## Materials and Methods

### Bacterial Plasmids, Strains, Culture Conditions

*Escherichia coli* strain TG1 (Zymo Research) were used as the host for plasmid cloning. *E. coli* strains containing pLysS i.e., BL21 (DE3) (GE Healthcare) or *E. coli* BL21 (DE3)-derivative, with the deletions of the *endA* and *recA* genes (Acella™, Edge Bio), were used for the production of the His_6_-tagged and glutathione-S-tranferase (GST)-tagged fusion proteins, respectively. Chromosomal DNA from *S. aureus* strain Newman was used to amplify *vwb* and *coa* genes. *E. coli* strain TG1 were grown on LB medium supplemented with ampicillin (100 μg/ml). *E. coli* BL21 (DE3)pLysS and Acella strains were grown on Terrific Broth medium (Invitrogen) supplemented with ampicillin (100 μg/ml) and chloramphenicol (35 μg/ml).

### Cloning of vWbp and Coa Constructs

Genomic DNA isolated from *S. aureus* strain Newman was used as the template for all PCR experiments with oligonucleotide primers described in Supplementary Table 1 of the supplementary figure, restriction enzyme cleavage sites are underlined. For His-vWbp and Coa, PCR products were digested with BamHI and PstI (New England BioLabs) and then ligated into pRSET-A vector (Invitrogen). For GST-vWbp, PCR products were digested with BamHl and EcoRI and ligated into pGEX-5x-1 (GE Healthcare). The ligation mixture for vWbp was transformed in *E. coli* strain TG1 cells (Zymo Research). Cloning of GST-Coa was described previously (Ko et al., 2016). These were grown on LB agar plates containing 100 μg/ml ampicillin to select for transformants. Insertions were confirmed by DNA sequencing.

### Expression and Purification of Recombinant Proteins

*E. coli* strains containing plasmids pRSET-A-vWbp/Coa or pGEX-5x-1-vWbp/Coa were grown overnight at 37°C in Terrific Broth medium containing 100 μg/ml ampicillin and 35 μg/ml chloramphenicol. The overnight cultures were then diluted 1:50 into fresh Terrific Broth medium, and grown to an OD_600_ of 0.8–1. Recombinant expression was then induced with 0.2 mM isopropyl β-D-1-thiogalactopyranoside (Gold Biotechnology, Inc.) for 3 h at 37°C. Bacteria were then harvested, centrifuged and lysed using the French press (SLM Aminco). Soluble recombinant proteins were purified by nickel chelate chromatography (GE Healthcare) and anion exchange chromatography (GE Healthcare) or through a glutathione -Sepharose 4B column (GE Healthcare) according to the manufacturer's manual.

### Gel Permeation Chromatography

The peak fractions of each recombinant protein from either affinity or ion exchange chromatography were pooled, concentrated and then further purified by gel permeation chromatography, using a HiLoad 16/600 Superdex 200 pg column (GE Healthcare) equilibrated in 1.2x Phosphate Buffered Saline (PBS; Gibco, pH 7.4). The recombinant proteins were then stored at −20°C. Protein concentration was determined by the Bradford assay (Pierce). Protein size and purity were analyzed on SDS-PAGE gels and stained with Coomassie Blue R-250 (Sigma Aldrich).

### Fibrinogen (Fg)

Human fibrinogen (Catalog # FIB3, Enzyme Research Laboratories) was used in all experiments.

### Circular Dichroism Spectroscopy (CD)

CD spectra were measured in far UV (185 nm−260 nm) to measure secondary structure composition. The instrument used was Jasco J720 spectropolarimeter that was calibrated with d-10-camphorsulfonic acid, with a band pass of 1 nm and integrated for 1 s at 0.2-nm intervals. His-vWbp or -Coa was measured at a concentration of 0.6–0.3 mg/ml in PBS, pH 7.4. Spectra were recorded at an ambient temperature in cylindrical 0.5 mm path length cuvettes. Ten scans were averaged for each spectrum and the contribution from the buffer was subtracted. Deconvolution software programs, BeStSel and CAPITO, were used to quantify the secondary structures (Wiedemann et al., 2013; Micsonai et al., 2018).

### ELISA Type Binding Assay

96-well immulon 4HBX (Thermo Fisher Scientific) microliter plates were used. Wells were coated with 100 μl of 5 μg/ml of either Fg (diluted in phosphate-buffered saline [PBS]; Gibco, pH 7.4) or GST-vWbp or -Coa (diluted in PBS), for overnight at 4°C. Plates were blocked with 3% BSA in TBS (25 mM Tris, pH 7.4, 3 mM KCl, and 140 mM NaCl). For immobilized Fg binding, diluted recombinant vWbp or Coa proteins (in 1% BSA, 0.05% Tween 20, TBS) were added to the Fg- coated wells and incubated for 1 h at room temperature. Bound vWbp or Coa proteins were detected using Horseradish Peroxidase (HRP)- conjugated anti-GST polyclonal antibodies (6,000X dilution) (Abcam). For soluble Fg binding, diluted Fg (in 1% BSA, 0.05% Tween 20, TBS) was added to vWbp- or Coa- coated wells. Bound Fg was detected using HRP-conjugated Human Fg polyclonal antibodies (10,000X dilution) (Rockland Immunochemicals, Inc.). Binding was quantified by the addition of substrate o-phenylenediamine dihydrochloride (Sigma-Aldrich) and the absorbance was measured at 450 nm using the ELISA microtiter plate reader (ThermoMax). Raw data was fitted using the one-site binding equation, apparent *K*_D_ values and goodness of fit (R^2^) were obtained from GraphPad Prism software version 4.0. Apparent *K*_D_ values represent averages of three independent experiments.

### Competition- Based ELISA Assay

96-well immunlon 4HBX microtiter plate was coated with full length Fg (5 μg/ml). Various concentrations of the GST-vWbp or -Coa were mixed with a fixed concentration of His- vWbp (200 nM) or -Coa (1 nM); Peptide Coa-RI or Efb-O (Shanghai Hanhong Scientific Co., Ltd.) (Ko et al., 2016) was mixed with a fixed concentration of GST- vWbp-C (500 nM) or -Coa-C (0.6 nM); Fg β-chain peptide (1-QGVNDNEEGFFSARGHRPLDKKREE-25) or scrambled Fg β-chain peptide (1-FSERKDLHQGEGNPREFVENDAKGR-25) (Shanghai Hanhong Scientific Co., Ltd.) (Ponnuraj et al., 2003) was mixed with a fixed concentration of GST-vWbp-N (6 nM) or -Coa-N (400 nM). All proteins and peptides were diluted (in 1% BSA, 0.05% Tween 20, and TBS) and were added to coated wells. Binding was detected using the HRP-conjugated rabbit-anti-his polyclonal antibodies (5000X dilution) (Alpha Diagnostic Intl. Inc.) or HRP- conjugated anti-GST polyclonal antibodies (6,000X dilution). Results were presented as percentage of binding to Fg and 100% binding Fg was defined as the absorbance measured in the absence of the inhibitor. Data was normalized to the % of binding Fg in the presence of the inhibitor. The data was then fitted using the one-phase exponential decay equation from GraphPad Prism software version 4.0. The competition experiments were carried out three times.

### Far Western

Fg (5 μg/lane) was reduced in Laemmli buffer (Bio-Rad) and separated on 10% SDS-PAGE gel. Reduced Fg was then stained with Coomassie Blue or electro transferred to a nitrocellulose membrane (Bio-Rad). Nitrocellulose membrane was blocked with 5% non-fat milk in TBS. Upon rinsing with blocking reagent, the membrane was probed with either recombinant vWbp-N (5 μg/ml), Coa-N (5 μg/ml), SdrG (2.5 μg/ml) or ClfA (15 μg/ml) (Vazquez et al., 2011). Membrane was incubated with proteins for 1 h at room temperature. After washing with TBST (0.1% Tween 20 and TBS), bound protein was detected with HRP-conjugated GST polyclonal antibodies (6000X dilution) or HRP-conjugated Rabbit-anti-His polyclonal antibodies (5000X dilution) (used for SdrG and ClfA), for 1 h at room temperature. Detection was developed using HyGLO Chemiluminescent reagent (Denville), according to manufacturer's manual.

## Results

### Recombinant vWbp and Coa Have Similar Secondary Structure Compositions

**Figure 1:**
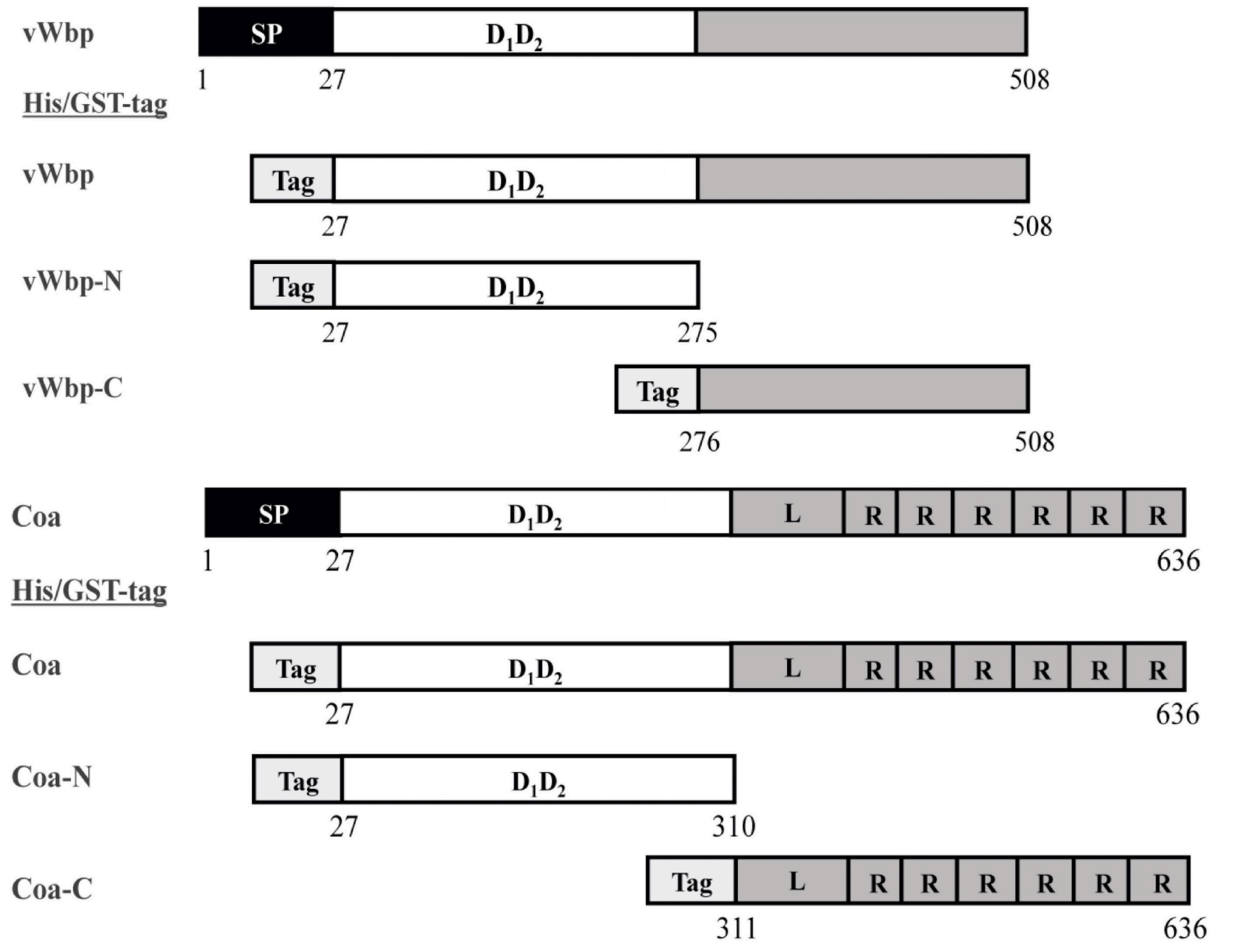
Constructs of the recombinant coagulases. Schematic overview of the vWbp and Coa constructs generated in this study. Gray represents the predicted unordered region. SP, Signal peptide; D_1_D_2_, prothrombin-binding domain. R, repeat domain; L, linker region. Tag box, His or GST-tag at N-terminal.

**Figure 2:**
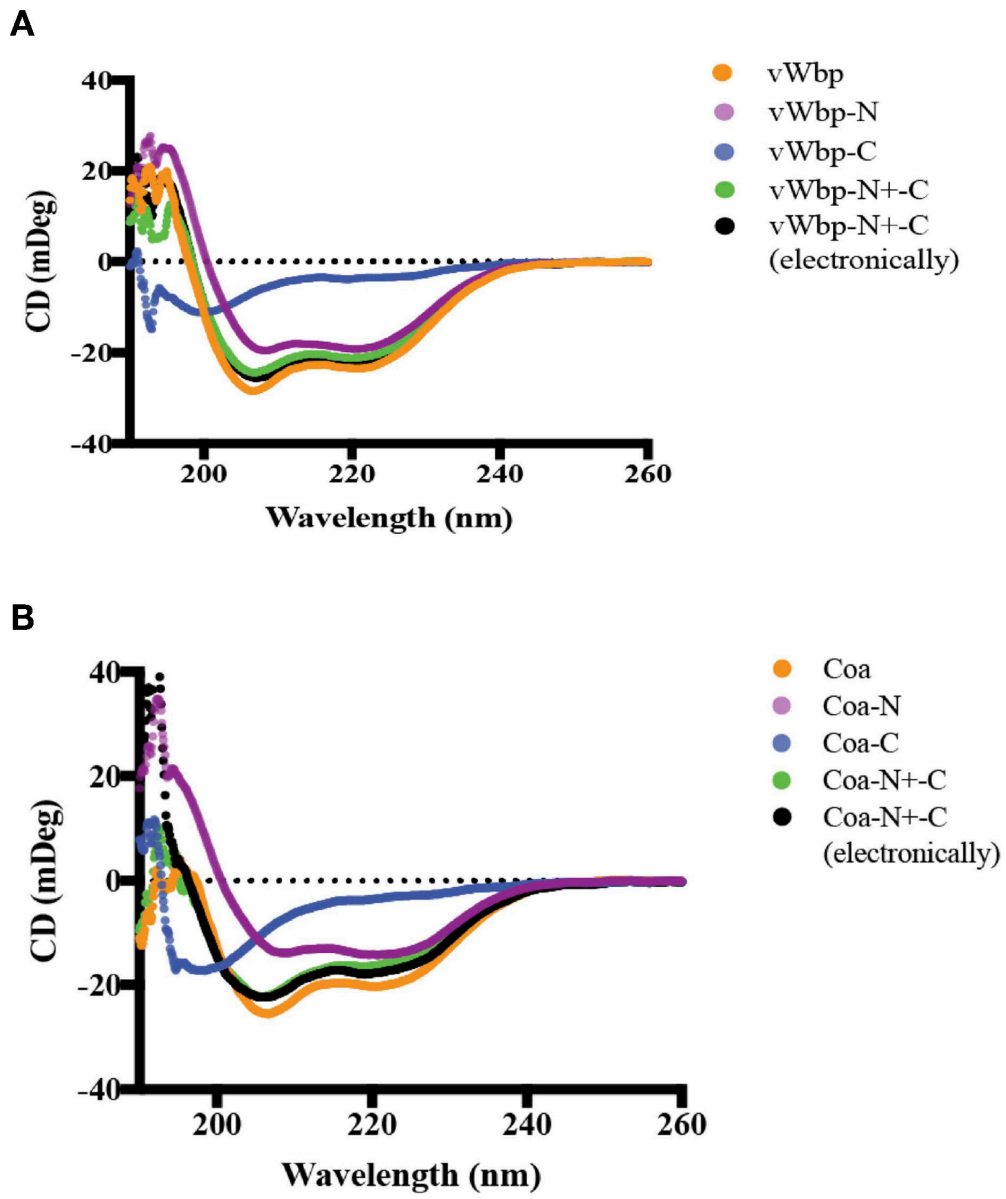
Circular dichroism analyses of secondary structures of vWbp and Coa**. (A)** vWbp (10 μM; orange), vWbp-N (9 μM; purple), vWbp-C (10 μM; blue). vWbp-N mixed with vWbp-C (5 μM; vWbp-N+-C, green). Sum of CD spectra of vWbp-N and vWbp-C using Excel (vWbp-N+-C electronically, black). **(B)** Coa (8 μM; orange), Coa-N (8 μM; purple), Coa-C (8 μM; blue). Coa-N mixed with Coa-C (4 μM; vWbp-N+-C, green). Sum of CD spectra of Coa-N and Coa-C using Excel (Coa-N+-C electronically, black). mDeg, millidegrees. The graphs are representative of the three independent experiments.

In order to characterize vWbp and Coa and their interactions with Fg, we expressed and purified recombinant versions of the full-length proteins, as well as, the N- and C-terminal halves with a His_6_-or a glutathione-S-transferase (GST) tag fused to the N-terminus (Figure 1). The protein purities were analyzed by SDS-PAGE and stained with Coomassie Blue (Supplementary Figures 1a,b). The secondary structures of the His-vWbp and Coa proteins were analyzed by circular dichroism (CD) [Figures 2A,B; (Kelly et al., 2005)]. The spectra for the vWbp proteins and the corresponding Coa proteins are similar (Figures 2A,B). This is also shown from the secondary structure composition data using deconvolution programs (Supplementary Figures 2a,b). The spectra of full-length vWbp and Coa show two negative bands at ~220 and 206 nm (Figures 2A,B, orange). The data suggests that the N-terminal regions for both vWbp and Coa are dominated by alpha helices, as shown by the signature alpha helical spectra of two negative bands near 222 and 208 nm [Figures 2A,B, purple; (Kelly et al., 2005)]. These results are in line with the crystal structure of Coa-N in complex with prothrombin, where the staphylococcal protein domain is composed of two, three-helix bundles (67% alpha helical, 13 helices) (Friedrich et al., 2003) and support the predicted structure of vWbp-N, which is proposed to be similar to that of Coa-N [Figures 2A,B; (Kroh et al., 2009)]. The spectra for both vWbp-C and Coa-C show that the proteins harbor unordered regions (Figures 2A,B, blue; Supplementary Figure 3). The overall CD spectra signatures of full-length vWbp and Coa (Figures 2A,B, orange) are very similar to those of their N-terminal halves (Figures 2A,B, purple). The spectra of the mixture of vWbp-N with vWbp-C, and the sum of the spectra of the two proteins (vWbp-N+-C; green and black, respectively) are similar to the spectra of full-length vWbp and vWbp-N (Figure 2A). This is also observed for Coa (Figure 2B). Together, the data demonstrate that the recombinant intact proteins and the two subdomains of vWbp and Coa are similar in secondary structure composition as determined by CD.

### vWbp and Coa Differ in Their Relative Binding to Immobilized and Soluble Fg

**Figure 3:**
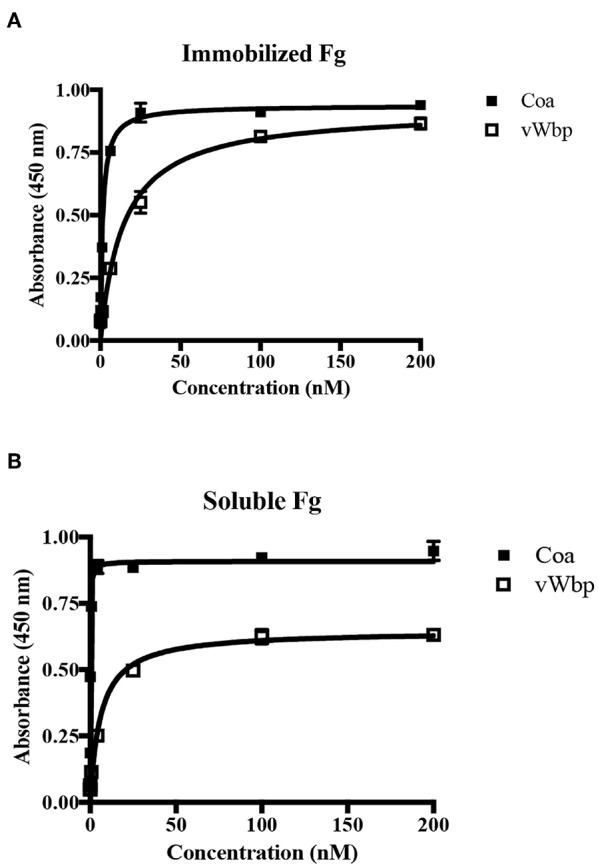
vWbp and Coa differ in their relative binding to immobilized and soluble Fg. **(A)** ELISA binding of GST-Coa or -vWbp to immobilized Fg (0.5 μg/well). **(B)** ELISA binding of Fg to immobilized GST-Coa or -vWbp (0.5 μg/well). Error bars, standard error of the mean (SEM). The graphs are representative of three independent experiments.

Under physiological conditions, Fg exists in multiple conformations, including a soluble form, which dominates when the protein is circulating in the blood, and an “immobilized” form, which Fg adopts when the protein is absorbed on the surface of an implanted device (Ugarova et al., 1993; Moreillon et al., 1995; Entenza et al., 2000). To explore how the coagulases interact with immobilized and soluble Fg, we used ELISA-type binding experiments. The binding results for Coa confirmed and extended earlier observations that the protein binds the two forms of Fg in a dose- dependent manner [Figures 3A,B; (Ko et al., 2016)]. Coa bound preferably to soluble Fg with almost a 30-fold higher apparent affinity compared to immobilized Fg (0.09 nM vs. 2.4 nM, Table 1) (Ko et al., 2016). vWbp also bound immobilized and soluble Fg, but did not show preference in affinity for either of the two forms of Fg (apparent *K*_D_ = 9.9 and 5.3 nM, respectively) (Figures 3A,B). As a negative control, neither vWbp nor Coa showed binding to wells coated with bovine serum albumin (BSA) (data not shown).

**Table 1:** Half-maximal binding concentrations (Apparent *K*_D_).

**Proteins**	**Constructs**	**vWbp**	**Coa**
Soluble Fg	Full-length	5.3 × 10^−9^ M ±	9.0 × 10^−11^ M ±
		8.9 × 10^−10^ M	6.1 × 10^−11^ M
Immobilized Fg	Full-length	9.9 × 10^−9^ M ±	2.4 × 10^−9^ M ±
		5.0 × 10^−9^ M	1.9 × 10^−9^ M
	N-terminal	3.2 × 10^−9^ M ±	2.0 × 10^−8^ M ±
		1.3 × 10^−9^ M	1.4 × 10^−8^ M
	C-terminal	3.8 × 10^−8^ M ±	4.6 × 10^−10^ M ±
		2.9 × 10^−8^ M	6.3 × 10^−11^ M

### vWbp and Coa Do Not Target the Same Binding Sites in Fg

**Figure 4:**
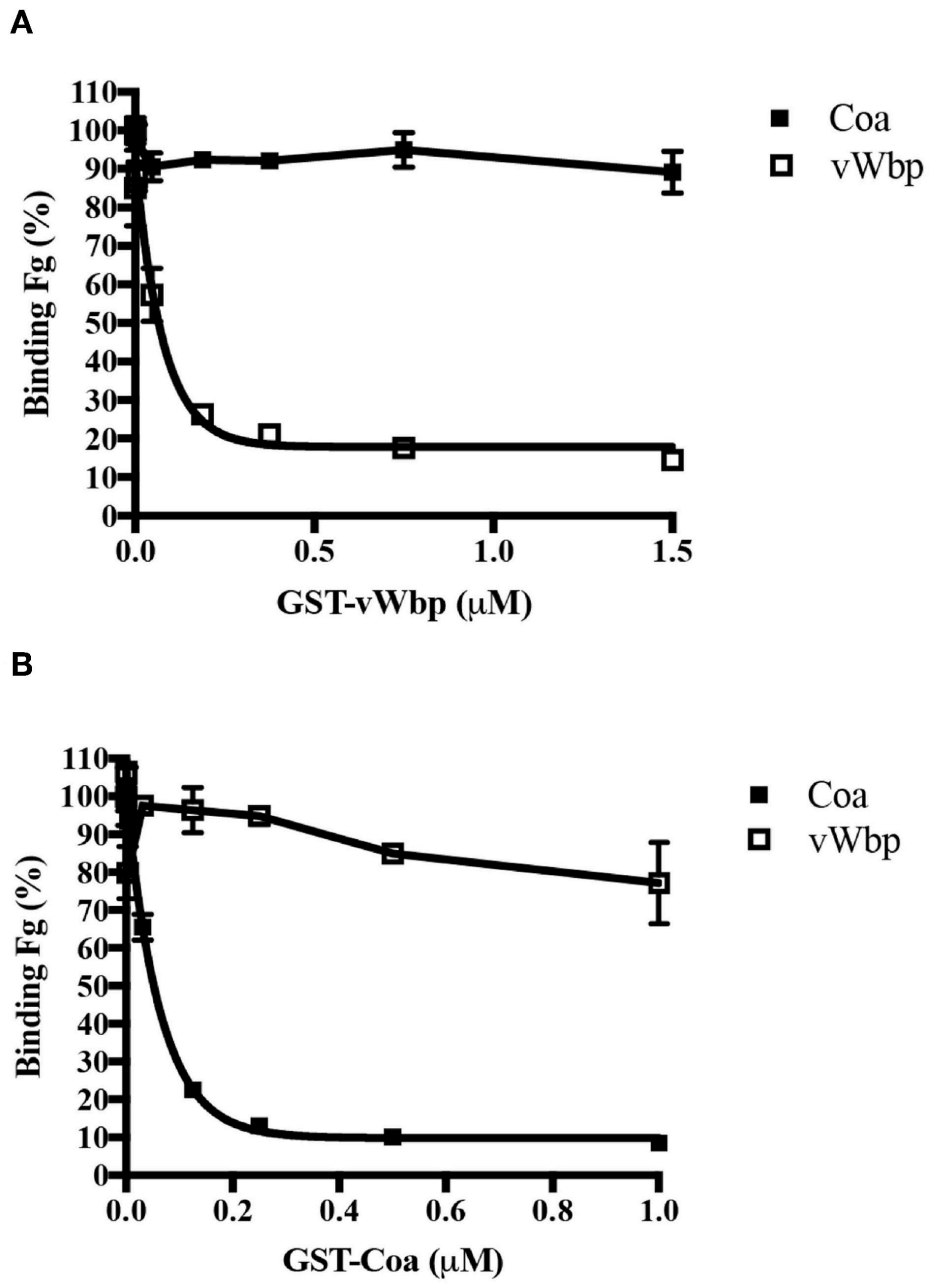
vWbp and Coa do not target the same binding sites in Fg. Competition ELISA of His-Coa (1 nM) or His-vWbp (200 nM) binding to immobilized Fg (0.5 μg/well) by **(A)** GST-vWbp and **(B)** GST-Coa. Error bars, standard error of the mean (SEM). The graphs are representative of three independent experiments.

Since both coagulases interact with Fg, we determined if vWbp and Coa target the same sites on Fg. To this end, we used a competition-based ELISA binding assay in which Fg was coated in a microtiter plate. The binding of His-vWbp or -Coa to immobilized Fg was evaluated in the presence of various concentrations of either GST-vWbp or -Coa. vWbp and Coa inhibited their respective selves from binding to Fg (Figures 4A,B). However, vWbp did not inhibit Coa from binding to immobilized Fg (Figure 4A), and Coa did not interfere with the binding of vWbp to Fg (Figure 4B, Supplementary Figure 4). Thus, these results suggest that vWbp and Coa bind Fg at different, non-overlapping sites.

### Both the N-Terminal and C-Terminal Halves of vWbp and Coa Bind Fg

**Figure 5:**
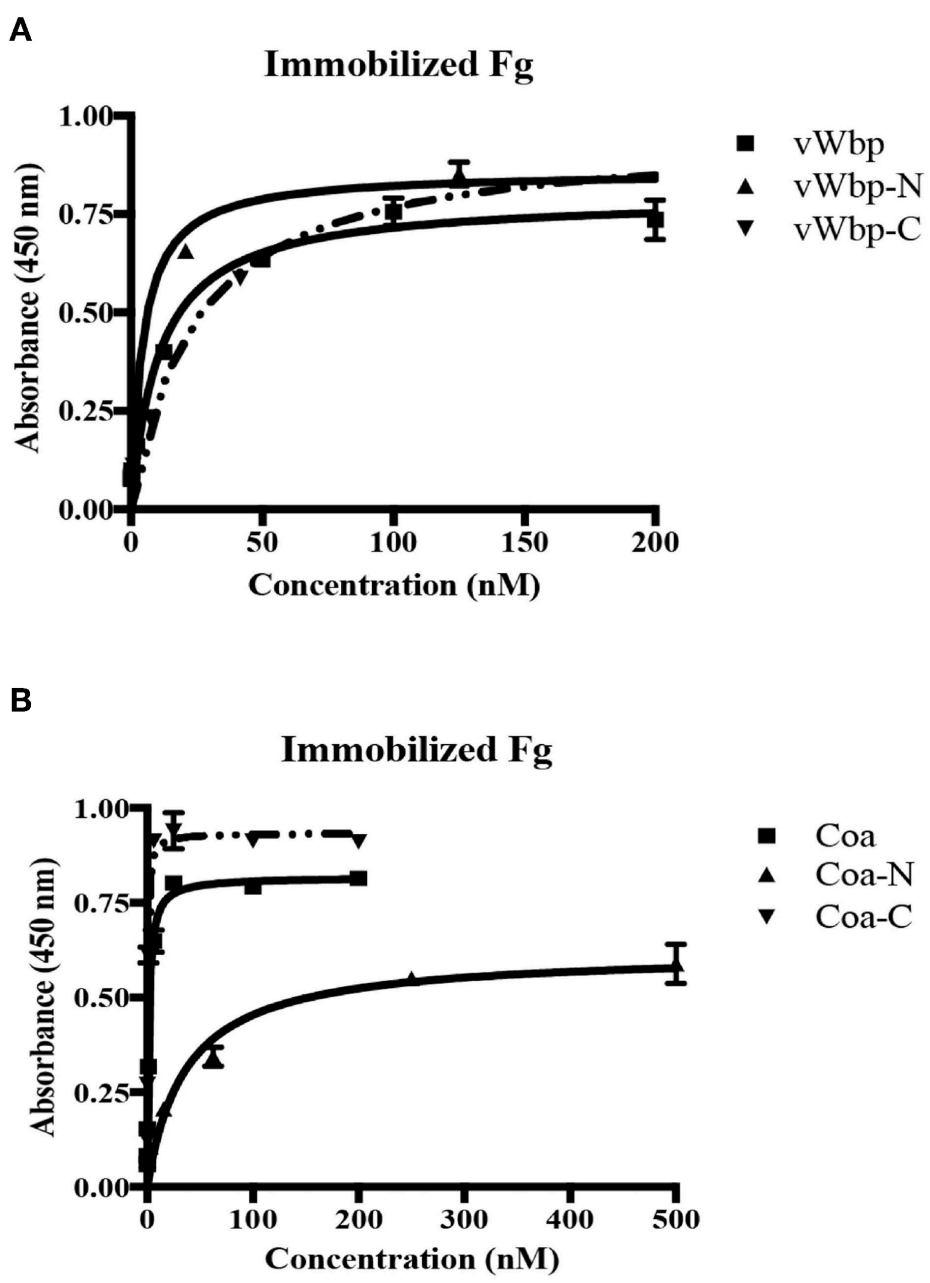
Both the N-terminal and C-terminal halves of vWbp and Coa bind Fg. **(A)** ELISA binding of GST-vWbp, -N, or -C to immobilized Fg (0.5 μg/well). **(B)** ELISA binding of GST-Coa, -N or –C to immobilized Fg (0.5 μg/well). vWbp or Coa (full length), vWbp-N or Coa-N (N-terminal), vWbp-C or Coa-C (C-terminal). Error bars, standard error of the mean (SEM). The graphs are representative of three independent experiments.

Although vWbp has been shown to bind Fg, the actual location of Fg-binding site(s) in vWbp remains to be defined (McAdow et al., 2012a; Thomer et al., 2013). To locate the Fg-binding site, we determined the Fg-binding activity of the N- and C-terminal regions of vWbp in detail and compared their binding activities to that of full-length vWbp, using ELISA-type binding assays. Full-length vWbp, vWbp-N and vWbp-C bound to immobilized Fg, exhibiting dose-dependent binding and saturation kinetics with apparent *K*_D_s of 9.9, 3.2, and 38 nM, respectively (Figure 5A; Table 1). Interestingly, vWbp-N appeared to bind Fg with an ~10-fold higher affinity than vWbp-C. We also analyzed Fg binding to intact Coa and its subdomains (Figure 5B; Table 1). In accordance with our earlier data, both the N- and C-terminal halves of Coa harbor Fg-binding activities, with the N-terminal region having a lower affinity to Fg than its C-terminal part (reported apparent *K*_D_ = 200 nM and 7.5 nM, respectively; Ko et al., 2016). The apparent *K*_D_ determined for the C-terminal region of Coa showed a significantly higher affinity for Fg compared to that determined for the C-terminal half of vWbp (apparent *K*_D_ = 0.46 nM vs. 38 nM, respectively). Thus, both Coa and vWbp harbor at least two Fg-binding sites located at their N- and C-terminal sections. However, the relative potencies of these sites seem to differ between the two coagulases. The N-terminal half of vWbp contains the predominant Fg-binding activity, whereas, the C-terminal half is the major Fg-binding region of Coa.

### vWbp-C and Coa-C Do Not Share Similar Fg-Binding Motifs

**Figure 6:**
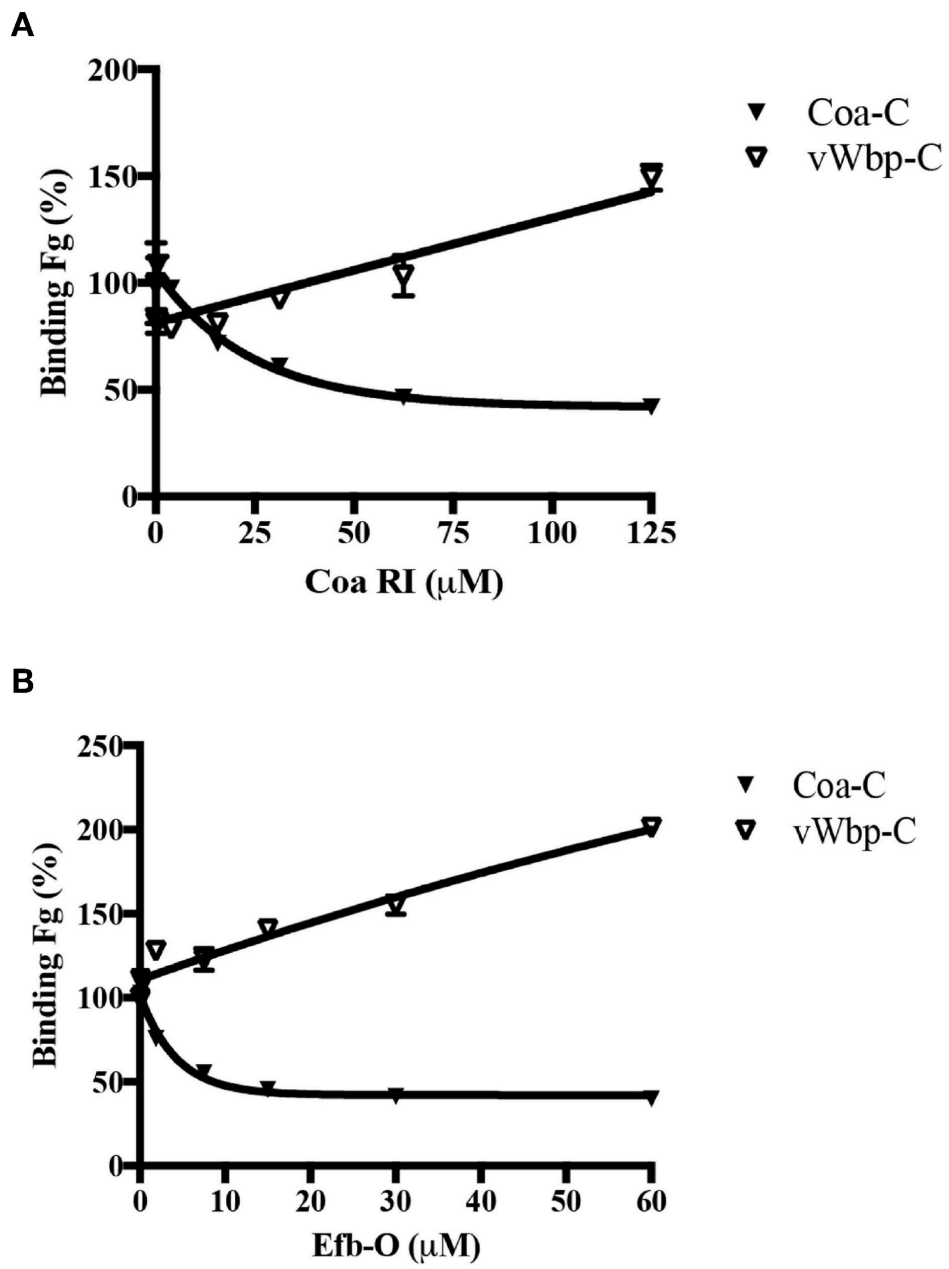
vWbp-C and Coa-C do not share similar Fg-binding motifs. Competition ELISA of Coa-C (0.6 nM) or vWbp-C (500 nM) binding to immobilized Fg (0.5 μg/well) by peptide **(A)** Coa RI and peptide **(B)** Efb-O. Error bars, standard error of the mean (SEM). The graphs are representative of three independent experiments.

Our earlier studies have shown that Coa and the secreted *S. aureus* protein Extracellular fibrinogen-binding protein (Efb) contain a common Fg-binding motif within their predicted disordered domains (Ko et al., 2011, 2016). Since vWbp-C is also predicted to be disordered, we wanted to determine if the Fg-binding site in vWbp-C is related to the Fg-binding motif in Coa or Efb. Using the synthetic peptides corresponding to the Fg-binding motif of Coa [Coa-RI; (Ko et al., 2016)] and Efb [Efb-O; (Ko et al., 2016)], we performed a competition-based ELISA-type assay using these peptides as potential inhibitors. We determined the binding of vWbp-C and Coa-C to immobilized Fg in the presence of various concentrations of Coa-RI or Efb-O (Figures 6A,B). As expected, Coa-C was inhibited from binding to Fg by both peptides. However, the Coa peptide (Figure 6A) and the Efb peptide (Figure 6B) did not inhibit vWbp-C binding to Fg, suggesting that vWbp does not harbor the Fg-binding motif found in Coa and Efb. Intriguingly, the increasing amount of peptide added enhanced vWbp-C binding to immobilized Fg. Since these peptides also bind Fg, it is possible that this binding results in a structural change to the protein, which accommodates more favorable vWbp-C binding. We, therefore, conclude that the disordered C-terminal halves of vWbp and Coa contain different Fg-binding motifs. Specifically, Coa contains the Efb-O type of binding motif, and vWbp contains a distinct and unidentified Fg motif.

### The N-Terminal Region of vWbp Binds to the N-Terminal of the Fg β-Chain

**Figure 7:**
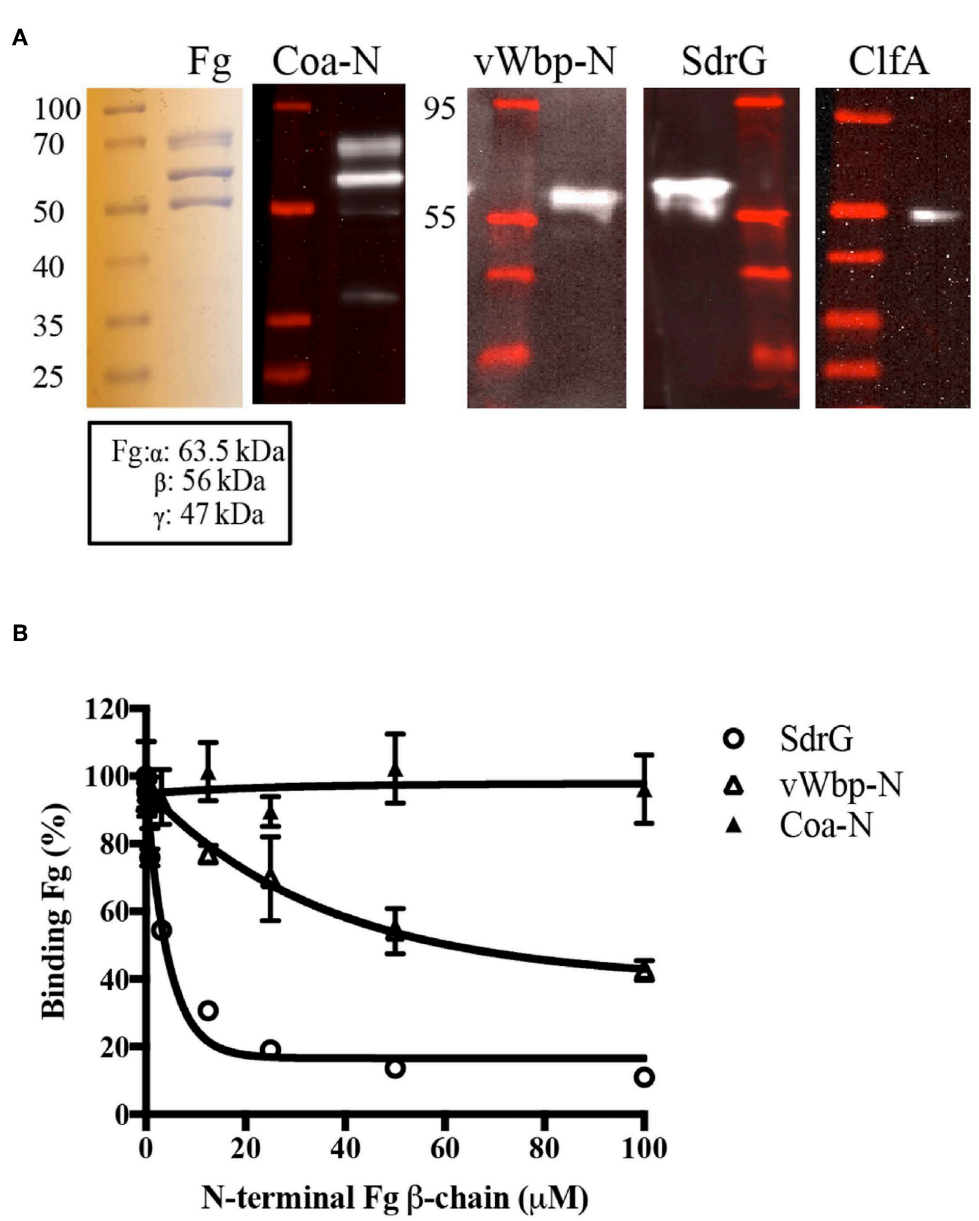
The N-terminal region of vWbp binds to the N-terminal of the Fg β-chain. **(A)** Far Western, Fg (5 μg/ lane) was separated on a SDS-PAGE gel and probed with vWbp-N (15 μg/ml) or Coa-N (5 μg/ml). ClfA (15 μg/ml) and SdrG (2.5 μg/ml) were used as controls. **(B)** Competition ELISA of SdrG (50 nM), vWbp-N (6 nM) or Coa-N (400 nM) binding to immobilized Fg (0.5 μg/well) by Fg β-chain (1-25) peptide. Error bars, standard error of the mean (SEM). The graphs are representative of three independent experiments.

We next wanted to characterize how vWbp-N interacts with Fg. In an attempt to identify the Fg polypeptides targeted by the recombinant vWbp-N and Coa-N, we used far western analysis. The three Fg polypeptide chains were separated by SDS-PAGE and transferred to a supporting membrane to be probed with vWbp-N and Coa-N, respectively (Figure 7, Supplementary Figure 6). ClfA and SdrG, two staphylococcal Fg-binding MSCRAMMs known to bind to the C-terminus of γ- chain (ClfA) and the N-terminal of the β-chain (SdrG), served as controls (McDevitt et al., 1997; Ponnuraj et al., 2003; Ganesh et al., 2008). The results revealed that both vWbp-N and Coa-N bound to the β-chain of Fg, but Coa-N also bound weakly to the α-chain. Since vWbp-N binds to the denatured form of the Fg β-chain, it is likely that vWbp-N binds to a specific linear sequence of the β-chain. SdrG was previously shown by our laboratory to bind to the first 25 amino acid residues of the Fg β-chain, which is unordered (Ponnuraj et al., 2003). We, therefore, examined if vWbp-N binds to this region, by using a synthetic peptide corresponding to the Fg β-chain 1-25 residues [Fg β (1–25)] in a competition-based ELISA assay, where Fg was coated in a microtiter plate. Binding of vWbp-N to immobilized Fg was evaluated in the presence of increasing concentrations of the peptide Fg β (1-25). As expected, SdrG, which served as the positive control, was effectively inhibited by the peptide Fg β (1-25) from binding to full length Fg (Figure 7B). A scrambled 25-residues peptide did not inhibit vWbp-N or SdrG binding and served as a negative control (Supplementary Figure 5). The results showed that the Fg β (1-25) peptide only partially inhibited vWbp-N from binding to Fg, suggesting that Fg β (1-25) constitutes a part of the binding site and that additional Fg residues beyond the Fg β (1-25) are likely involved in the interaction. In contrast, binding of Coa-N to full-length Fg was not affected by the Fg β (1-25) peptide, suggesting that Coa recognizes a different region in the Fg β-chain.

## Discussion

In this study, we have compared the secondary structure compositions and the Fg binding of the *S. aureus* coagulases Coa and vWbp. The similarity in circular dichroism (CD) spectra of the N-terminal halves of the two coagulases support earlier sequence analyses and molecular modeling studies (Friedrich et al., 2003; Kroh et al., 2009; Kroh and Bock, 2012; Ko et al., 2016; Liesenborghs et al., 2018). This suggests that it is likely that the D_1_D_2_ domain of vWbp-N, composed primarily of alpha helices, resembles the structure of the corresponding Coa segment (Friedrich et al., 2003), despite the fact that these segments only have 30% sequence identity (Bjerketorp et al., 2004). In addition, our CD data showed that substantial sections of the C-terminal regions of both vWbp and Coa have an intrinsically disordered character (Figures 2A,B, Supplementary Figure 3).

vWbp and Coa fall into the zymogen activator and adhesion protein (ZAAP) family since they activate prothrombin non-proteolytically (Friedrich et al., 2003; McAdow et al., 2012b). Both coagulases can induce clotting of murine blood (Bjerketorp et al., 2004; Cheng et al., 2010) and the observed effects of vWbp and Coa in mouse infection models therefore seems clinically relevant. At the nidus of a murine abscess model, *S. aureus* is surrounded by a pseudocapsule (Cheng et al., 2009; McAdow et al., 2011), a layer of Fg that also contains Coa and prothrombin as revealed by antibody staining (Cheng et al., 2010). The periphery of the pseudocapsule is composed of another layer of Fg/fibrin that exhibits a pronounced staining of vWbp. This suggested that vWbp predominantly co-localizes with Fg, as well as prothrombin, at this second fibrin layer. The distinct two layers of Fg structure observed in the murine abscess model is re-established in the 3D-Collagen-Fg system, in which *S. aureus* microcolonies assemble two concentric Fg/fibrin barriers (Guggenberger et al., 2012). In this *in vitro* culture model, Coa is found to contribute to the formation of the pseudocapsule that encloses the microcolonies; whereas, vWbp is critical to the formation of the Fg/fibrin meshwork, which lies immediately adjacent to the pseudocapsule (Guggenberger et al., 2012). These observations suggest that despite sharing similar secondary structures and interacting with Fg and prothrombin, the coagulases contribute differently in assembling Fg/fibrin shield structures. Furthermore, the two coagulases reportedly differ in the role that Fg-binding plays in prothrombin activation. Fg enhances vWbp but not Coa-mediated prothrombin activation (Kroh et al., 2009).

Our study reports significant differences both in the Fg-binding affinities of these two coagulases and in the sites of interaction between the coagulases and Fg. We showed that both bacterial proteins recognize both soluble and adsorbed Fg, which represent two different conformations of the host protein, yet only Coa seems to prefer the soluble over the immobilized form of Fg (Figures 3A,B; Table 1). The observed difference could be the consequence of a conformational change in Fg induced on binding to Coa. Restricted mobility for the adsorbed form of Fg could limit this flexibility. In this scenario, soluble Fg but not adsorbed Fg could undergo a hypothetical conformational shift associated with high affinity binding of Coa to Fg. On the other hand, vWbp showed essentially no preference for either form of Fg, indicating that a similar hypothetical conformational restriction issue does not apply to Fg in its interaction with vWbp (Table 1). Furthermore, we noticed that the presence of either of the previously identified Coa or Efb Fg-binding peptides enhanced Fg binding to vWbp-C (Figures 6A,B). It is possible that the binding of the peptides to Fg induces a conformational change in the host protein resulting in an increased number and/or affinity of vWbp binding sites in Fg. Earlier studies suggest that vWbp undergoes a conformational change upon the binding to prothrombin (Kroh et al., 2009; Pozzi et al., 2013). These authors speculate that vWbp exhibits a certain degree of conformational plasticity. Our data appears to support this concept and also extends it to Coa. Our CD spectra analyses suggest that large sections of the C-terminal halves of both vWbp and Coa are intrinsically disordered (Figures 2A,B, Supplementary Figure 3). Intrinsically disordered regions exist in various conformational states and contribute to the structural flexibility of the protein (Portman, 2010; van der Lee et al., 2014). We also noticed significant batch-to-batch variations for both coagulases in the inhibition experiments, which are consistent with a mixture of conformational forms with different properties. Thus, it appears that the conformations of both Fg and the coagulases can be affected through their interactions. To fully understand how these virulence factors activate prothrombin, a detailed understanding of the conformational isoforms of the proteins involved and their transitions are needed.

The two coagulases each harbor at least two distinct Fg-binding sites located at the N-and C-termini segments of the proteins (5A,B). Under the experimental conditions used, the N-terminal domain (vWbp-N) is the predominant Fg-binding region in vWbp, and the C-terminal section (Coa-C) is the major Fg-binding region in Coa. We have previously identified a Fg-binding repeated linear motif in Coa-C (Ko and Flick, 2016; Ko et al., 2016), which is also present in the Fg-binding protein Efb (Ko et al., 2016). Sequence analysis indicates that vWbp does not contain this motif and consistent with this observation, synthetic peptides corresponding to the Coa and Efb versions of the motif, respectively, did not inhibit vWbp-C from binding to Fg (Figures 6A,B). Furthermore, the interactive sites in Fg targeted by the two coagulases are not shared. We demonstrated that vWbp-N binds to a synthetic peptide corresponding to the N-terminus of the Fg β-chain. Although Coa-N also appears to target the Fg β-chain, the protein appears to bind to a different site since the Fg β (1-25) peptide does not affect Fg binding to this segment of Coa. Thus, we conclude that the two coagulases make multiple connections with Fg but the interactive sites in both the host protein and the bacterial binding partner differ for vWbp and Coa, respectively.

Earlier studies on the interaction between vWbp and Fg, apparently gave conflicting results. McAdow et al. (2012a) showed using an ELISA-based approach that the Fg-binding activity was located in the C-terminal half of vWbp (McAdow et al., 2012a). The N-terminal half of vWbp binding to Fg was not determined in their study. Later, the same group used affinity chromatography (Thomer et al., 2013) where they found that the N-terminal region of vWbp, but not the C-terminal half, bound Fg. Our ELISA data presented here provides a potential explanation for the earlier published results. Both the N-and C-terminal regions of vWbp harbor Fg-binding sites. These observations paired with the different methods used in the earlier binding studies could possibly explain the contradicting results. In addition, coupling the disordered C-terminal segment of vWbp to the column may have reduced its conformational freedom; thereby, the Fg-binding region may be buried within the protein and not accessible for Fg to bind (Chang et al., 2007; van der Lee et al., 2014). Utilizing the ELISA based approach as a common method allowed us to analyze the binding of vWbp and Coa to both Fg adsorbed to a surface and Fg in a soluble state, and to compare the Fg-interaction with the subdomains of the two bacterial proteins.

The two coagulases of *S. aureus* have a similar structural organization and are able to activate prothrombin without cleaving the zymogen, but our data shows that in terms of their Fg interactions, the two proteins behave differently. Their interactions with Fg are complex, engage multiple contact sites, and appear to involve significant conformational changes in the interacting proteins, which may be essential for how they can act as critical virulence factors in different steps of the pathogenic process of *S. aureus* infections. Due to the differences noted between Coa and vWbp in their interaction with Fg, targeting one coagulase alone may not be an effective strategy for drug development. However, inhibiting the interactions of both coagulases could effectively block the protective Fg/fibrin shield assembly and serve as a preventive measure.

## Author Contributions

ST, WL, SA, VG, Y-PK, and MH conceived and designed the experiments. ST and WL performed the experiments. ST, Y-PK, and MH analyzed the data. ST, Y-PK, and MH wrote the paper.

### Conflict of Interest Statement

The authors declare that the research was conducted in the absence of any commercial or financial relationships that could be construed as a potential conflict of interest.
