# Supplementary material for: The Complex Fibrinogen Interactions of the Staphylococcus aureus Coagulases
Source: Front Cell Infect Microbiol. 2019 Apr 16;9:106. doi: 10.3389/fcimb.2019.00106 (PMC6476931; doi:10.3389/fcimb.2019.00106)
Supplement: Supplementary file 1 [file Data_Sheet_1.pdf]

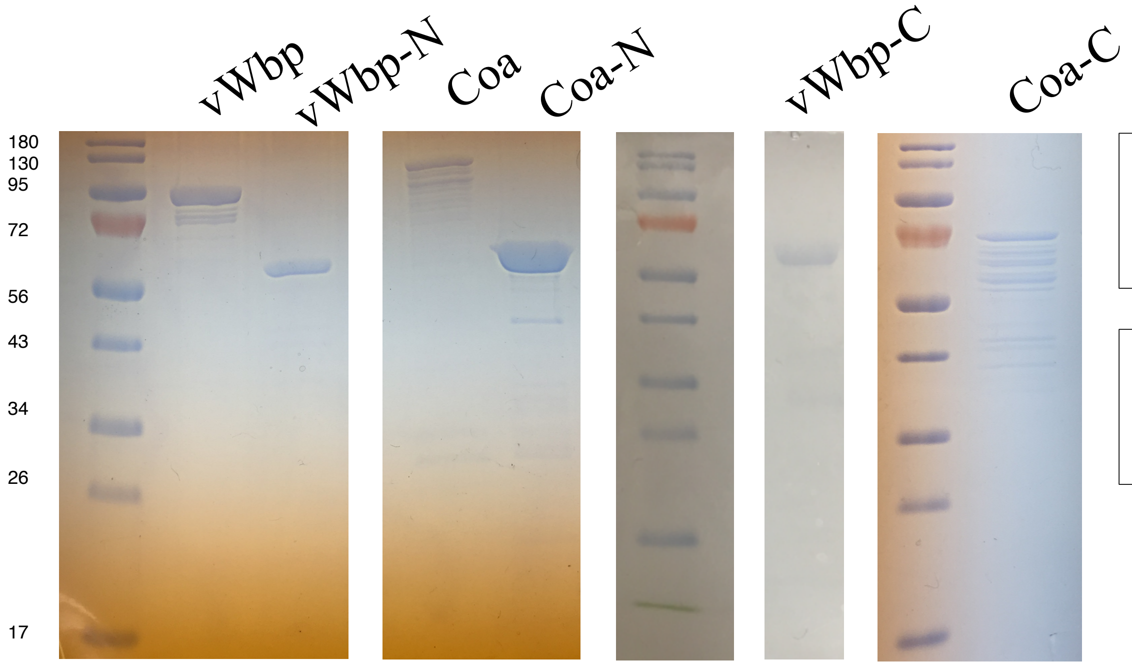


| Key: | (MW kDa) |
|---|---|
| vWbp | (83) |
| vWbp-N | (56) |
| vWbp-C | (53) |

| Key: | (MW kDa) |
|---|---|
| Coa | (95) |
| Coa-N | (60) |
| Coa-C | (62) |

**Supplementary Figure 1a. Coomassie stained gel of GST-tagged constructs.** C-terminal vWbp and Coa proteins show multiple bands, suggesting extensive degradation.

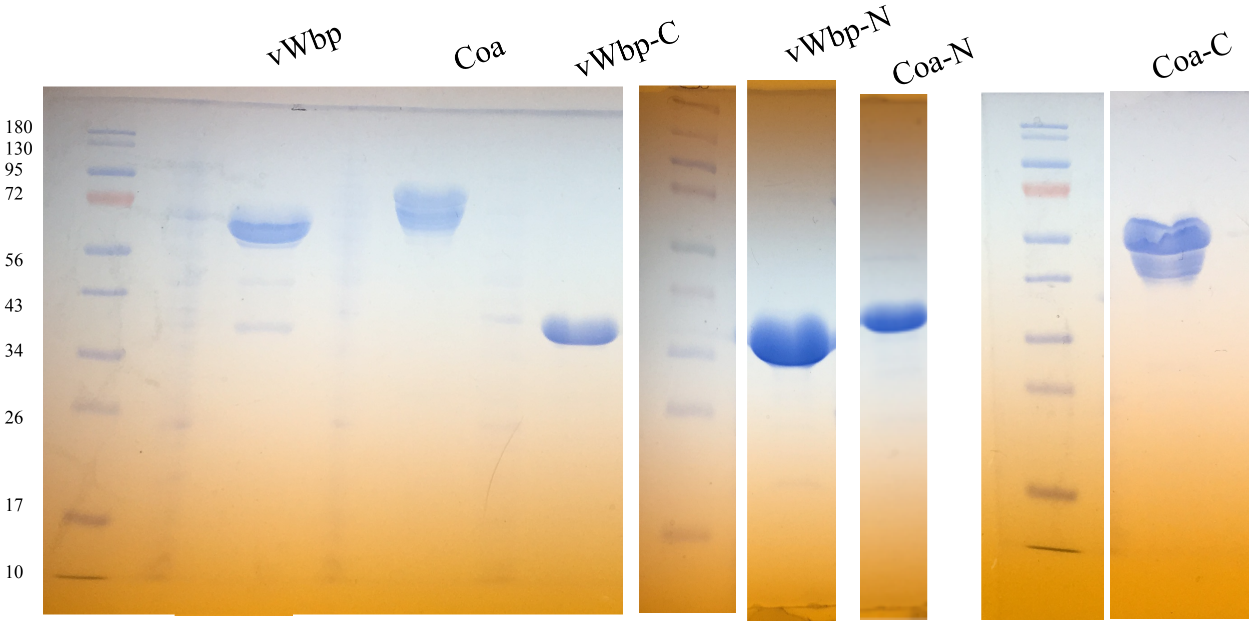


| Key: | (MW kDa) |
|---|---|
| vWbp | (60.6) |
| vWbp-N | (33.7) |
| vWbp-C | (31) |

| Key: | (MW kDa) |
|---|---|
| Coa | (73.1) |
| Coa-N | (37.7) |
| Coa-C | (39.6) |

**Supplementary Figure 1b. Coomassie stained gel of His-tagged constructs**

| Protein | Algorithm | α-helix | Parallel β-sheet | Anti-Parallel β-sheet | Turns | Unordered | Others |
|---|---|---|---|---|---|---|---|
| vWbp | Bestsel | 26.2 | 2.3 | 17.6 | 13.9 | | 40 |
| | CAPITO | 18.8 | 15 | | | 57.2 | |
| vWbp-N | Bestsel | 34.4 | 3.6 | 14.9 | 12.3 | | 34.8 |
| | CAPITO | 26.8 | 11.8 | | | 52 | |
| vWbp-C | Bestsel | 1.4 | 0.7 | 41.7 | 16.6 | | 39.8 |
| | CAPITO | 4 | 38.2 | | | 61.6 | |
| vWbp-N+-C | Bestsel | 32 | 3.6 | 11.3 | 13.5 | | 39.7 |
| | CAPITO | 29.5 | 6 | | | 59 | |

**Supplementary Figure 2a. Deconvolution of vWbp**

| Protein | Algorithm | α-helix | Parallel β-sheet | Anti-Parallel β-sheet | Turns | Unordered | Others |
|---|---|---|---|---|---|---|---|
| Coa | Bestsel | 20.4 | 3.7 | 18.6 | 14.8 | | 42.6 |
| | CAPITO | 15.8 | 16 | | | 60.4 | |
| Coa-N | Bestsel | 32.2 | 13.8 | 5.1 | 12.7 | | 36.2 |
| | CAPITO | 28.7 | 15 | | | 50 | |
| Coa-C | Bestsel | 5.3 | 0.08 | 42.7 | 16.2 | | 35.8 |
| | CAPITO | 6.8 | 21.5 | | | 72.5 | |
| Coa-N+-C | Bestsel | 30.8 | 3.1 | 12.5 | 13.2 | | 40.6 |
| | CAPITO | 27 | 5 | | | 63.5 | |

**Supplementary Figure 2b. Deconvolution of Coa**

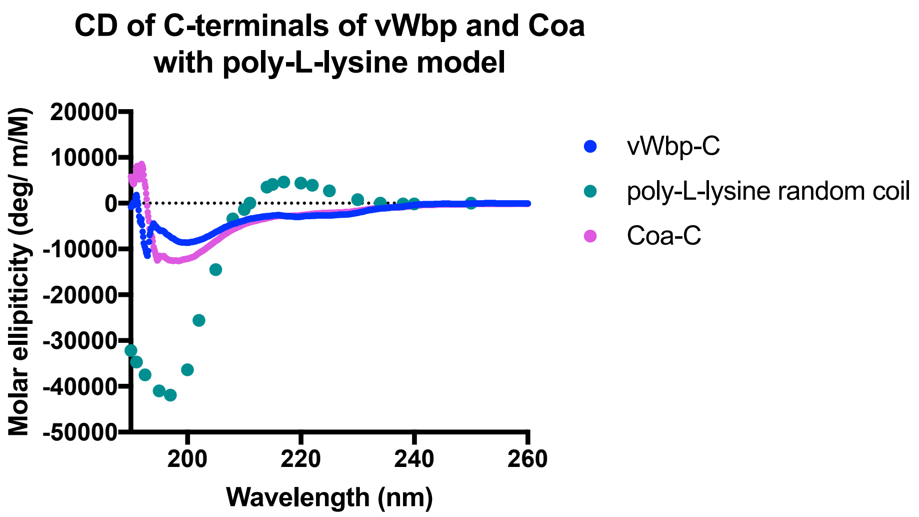


**Supplementary Figure 3. Circular dichroism of C-terminals vWbp and Coa**. Molar ellipiticity = deg x pathlength (mm) x concentration of peptide bonds (μM). Model used was poly-L-lysine, random coil structure (Greenfield and Fasman, 1969).

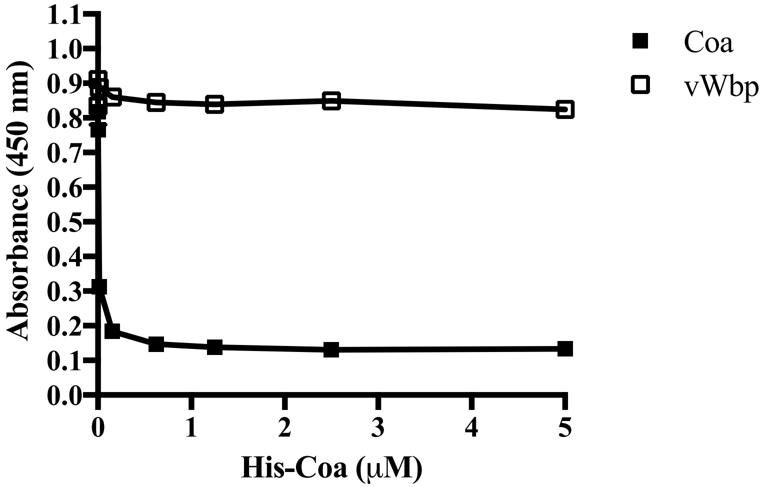


**Supplementary Figure 4. vWbp and Coa do not target the same binding sites in Fg**. Competition ELISA of GST-vWbp (25 nM) or GST-Coa (1 nM) binding to immobilized Fg (0.5 μg/well) by His-Coa. Error bars, standard error of the mean (SEM).

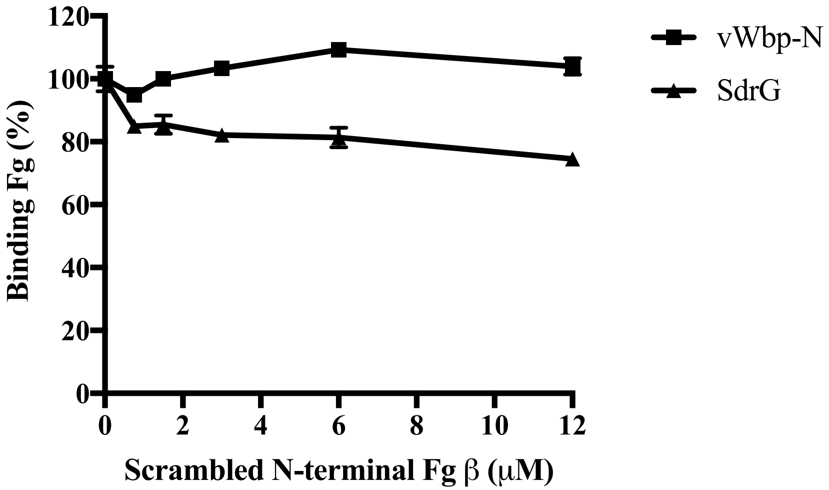


**Supplementary Figure 5. vWbp and SdrG do not bind to Scrambled Fg β peptide**. Inhibition of GST-vWbp-N (2 nM) or SdrG (50 nM) binding to immobilized Fg (0.5 μg/well) by scrambled Fg β (1-25) peptide. Error bars, standard error of the mean (SEM).

**Supplementary Table 1. Sequence of oligonucleotides used in this study**

| Primers | Forward sequence | Reverse sequence |
|---|---|---|
| His-vWbp | 5'- CAAG GGA TCC GTG GTT TCT GGG GAG AAG AAT C | 5'-CAAT C TG CAG TTA TTT GCC ATT ATA TAC TTT ATT GAT TTG |
| His-vWbp-N | 5'- CAAG GGA TCC GTG GTT TCT GGG GAG AAG AAT C | 5'- CAAT C TG CAG TTA TTC ATC ACT TTT TGC TGC TTC |
| His-vWbp-C | 5'- CAAG GGA TCC TCA AAA AGA AGC AAG AGA AG | 5- CAAT C TG CAG TTA TTT GCC ATT ATA TAC TTT ATT GAT TTG |
| GST-vWbp | 5' -CAA GGG ATC CCC GTG GTT TCT GGG GAG AAG AAT C | 5' -CAAT GAA TTC TTA TTT GCC ATT ATA TAC TTT ATT GAT TTG |
| GST-vWbp-N | 5' CAA GGG ATC CCC GTG GTT TCT GGG GAG AAG AAT C | 5' -CAAT GAA TTC TTA TTC ATC ACT TTT TGC TGC TTC |
| GST-vWbp-C | 5' CAA GGG ATC CCC TCA AAA AGA AGC AAG AGA AG | 5' -CAAT GAA TTC TTA TTT GCC ATT ATA TAC TTT ATT GAT TTG |
| His-Coa-full/-N/-C | (Ko et al., 2016) | (Ko et al., 2016) |
| GST-Coa-full/-N/-C | (Ko et al., 2016) | (Ko et al., 2016) |

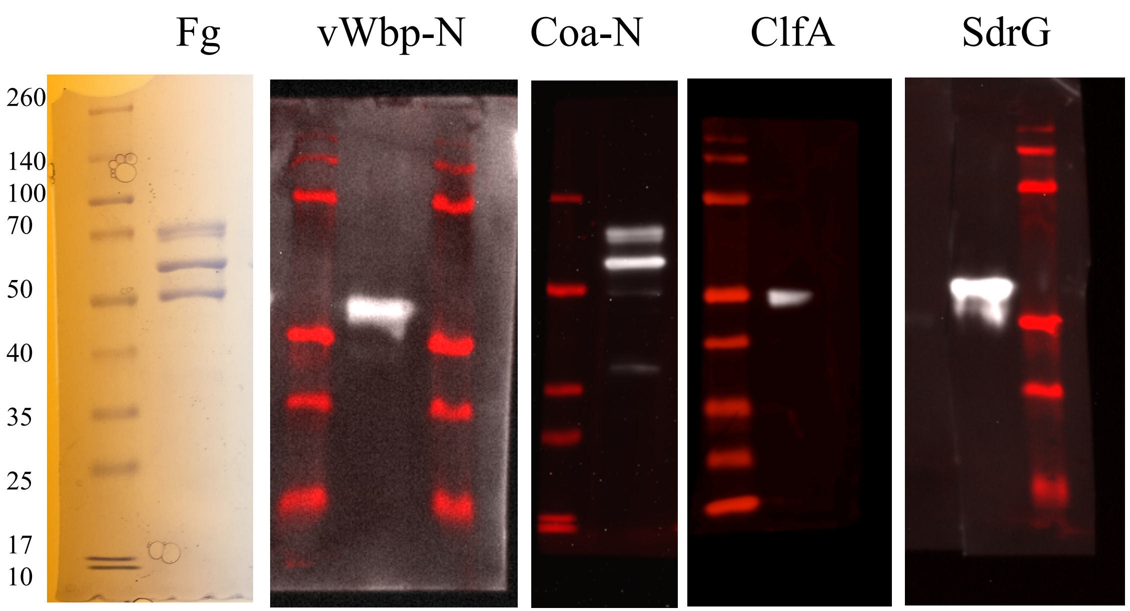


**Supplementary Figure 6. The N-terminal region of vWbp binds to the N-terminal of the Fg β-chain**. Far Western, Fg (5 µg/ lane) was separated on a SDS-PAGE gel and probed with vWbp-N (15 µg/ml). ClfA (15 µg/ml) and SdrG (2.5 µg/ml) were used as controls.
